# Treatment of Infected Wounds

**Published:** 1907-08

**Authors:** Emory Lanphear

**Affiliations:** St. Louis, Mo., Professor of Operative Surgery and Clinical Surgery in the Hippocratean College of Medicine


### TEXAS MEDICAL JOURNAL.

Established July, 1885.

F. E. DANIEL, M. D., - - - - Editor, Publisher and Proprietor

PUBLISHED MONTHLY.—SUBSCRIPTION $1.00 A YEAR.

VOL. XXIII. AUSTIN, AUGUST, 1907. No. 2.

The publisher is not responsible for the views of contributors.

For Texas Medical Journal.

### Treatment of Infected Wounds.

BY EMORY LANPHEAR, M. D., PH. D., LL. D., ST. LOUIS, MO.,
Professor of Operative Surgery and Clinical Surgery in the Hippocratean College of Medicine.

Every wound received accidentally is an infected one. Wounds inflicted by a surgeon should, theoretically, be aseptic, free from infection; but, unfortunately, many are not so because of (1) dirty fingers, (2) unclean instruments, (3) non-sterile gauze, (4) failure to drain when (a) the patient is very fat, (b) there is much oozing, (c) dead spaces have been left by unskillful suturing, or (d) the skin was not properly prepared, so that stitch abscesses have formed with subsequent general infection of the wound.

The first principle of treatment of an infected wound is free drainage. Therefore, contaminated wounds should not be closely sutured, save in the scalp where the excessive vascularity permits healing to occur more speedily than elsewhere. If closed without drainage, extensive suppuration is almost certain to occur, often with disastrous results—erysipelas, septicemia and lockjaw.

1. *Simple Incised Wounds.*—No amount of scrubbing, irrigation or application of antiseptic agents can transform an infected wound into a sterile one, which may be closed completely. Nevertheless, efforts at attaining "near asepsis" should be made—varying according to the character and location. When the wound is a clean cut—like that of a razor, sharp knife, sickle or other weapon that is not presumed to be reeking with bacteria, though not surgically clean, the proper treatment is to scrub the surrounding surfaces and the edges of the incision with soap and water, then wash with alcohol for a half minute and finally apply gauze saturated with solution of bichloride of mercury 1 to 2000 or phenol 1 to 40; finally flushing the wound itself with the sublimate or phenol solution.

In the absence of these agents turpentine makes a fairly good antiseptic agent.

A wound thus prepared is in faily good shape for closure. Very small wounds may be closed without drainage, as may also practically all cuts about the scalp and face. But in every other part of the body some kind of drain must be inserted. In many instances, if hemorrhage is completely controlled, two or three strands of catgut may be pushed to the depths of the wound at one or two places (according to size of cut) and left projecting through the skin; if there be much oozing, or the cut be deep, it is better to put in a little strip of gauze in one or two places.

For this partial closure of an infected wound, silkworm gut is the ideal suture, except for the face and scalp, where ten-day chromicized catgut No. 1 is to be preferred.

When the wound has thus been cleaned, closed and drained, it should be covered by several layers of gauze, preferably wrung out of the sublimate or phenol solution; a pad of absorbent cotton applied; and a bandage so placed as not to press tightly upon the wound, as this would interfere with the free drainage desired.

The closure of such wounds by collodion can not be too strongly condemned. The application of dusting powders, like iodoform, bismuth, etc., is also highly objectionable. Such wounds should be dressed on the third or fourth day. The gauze removed, the wound-surface is first carefully inspected and if found free from inflammation or much discharge the drains are withdrawn by sterile forceps and the surfaces quickly covered with gauze, without handling or washing. If there be much discharge the surface must be cleaned by gently wiping with clean gauze or cotton and the antiseptic dressing applied. If inflammation of a severe degree be deemed impending, some of the sutures may be cut and the wound permitted to gape. In some cases the wound should be inspected again in forty-eight hours.

*Badly Lacerated Wounds.*—The method of cleaning of a badly lacerated wound is the same as that of the simple wound except as to the management of the wounded surfaces themselves.

If there be grease and much dirt in the wound it is a good plan to clean it out with gasoline, followed by 65 per cent alcohol and then 1 to 2000 sublimate solution. All scraps of injured skin, muscle, etc., which are so injured as to be certain to die must be trimmed away with scissors—leaving them only makes infection worse and delays healing many days.

It is in these deep, lacerated wounds that antiseptics like iodoform do good. A very useful preparation is

Camphor ........................39 parts.
Phenol ..........................21 parts.
Liquid petrolatum..................40 parts. Mix.

This may be poured into the wound freely, the excess being permitted to run into the gauze which is applied over the hole. It is especially valuable in wounds involving joints.

Or if the bone be injured it is usually better to fill the depths with iodoform and cover with 10 per cent iodoform gauze.

Sometimes the wound may be partially closed by stitches—patients prefer to have it so; but often it is best just to pack the cavity loosely with sterile gauze.

This kind of a wound should be dressed in forty-eight hours, or sooner if the gauze and cotton become saturated with serum and pus or if high fever develop. At the first dressing it is best not to disturb the depths unless there be fragments of tissue to remove or necessity for providing freer drainage.

At the second or third dressing wound secretion will be abundant. If putrefaction is going on there will be a disagreeable "stinking" odor, and it may be necessary to trim out dying tissue and pour in a considerable quantity of liquid phenol (*i. e.*, 95 per cent carbolic acid), almost instantly neutralizing it by pouring in pure alcohol. Then the dressings are applied as before.

After a few days the amount of discharge may be very great, from a large wound; so great that the surgeon desires to lessen it. For this purpose may be used

Resorcin ..........................1 part.

Boric acid........................20 parts.

Apply freely to all parts of the infected surfaces and cover with gauze and cotton.

The practice of covering the dressings of these wounds with rubber tissue or oiled silk can not be too severely criticized. Such "protective" simply adds to the ability of the bacteria to multiply.

In some cases, when granulation seems to be delayed too long, it is good practice to cover the wounded surfaces with a few layers of bichloride gauze held in place by strips of adhesive plaster around the edges and place the patient where the wind may blow over the sore; or in winter to place the patient near a fire so that by evaporation of the watery part of the discharge the process of Nature's "healing under a scab" may be simulated.

After granulation has been well begun the less the surface is disturbed the better. Dressings should be changed only when becoming foul, and the discharges must be merely mopped away in the gentlest manner possible—all water, hydrogen dioxide, sublimate solution, etc., should be banished, save for cleaning the skin contiguous to the wound, and, as a rule, the less that is disturbed, also, the better. Plenty of clean gauze and cotton, with a loose bandage and perfect quietude will now do more than all the antiseptics made. It is hard to make the average nurse leave such wounds alone—she invariably wants to "do something," and often the patient and doctor also do. "Blessed is he who has learned well to do nothing."

3. *Punctured Wounds.*—Punctured wounds are extremely dangerous, since they are always infected and do not by their nature permit drainage without enlargement by the surgeon. Penetrating wounds of the abdomen are particularly serious, and the invariable rule should be to enlarge and explore them or to make a median section if viscera are known to be injured.

Every penetrating wound of any great magnitude should be treated by thorough cleaning of the adjacent skin and wide incision to the bottom of the original injury, however deep it may be. Then, unless some internal organ or delicate tissue prohibit, the surface may be cauterized with pure phenol, followed in half a minute by pure alcohol. Before this is done, however, careful search should be made for foreign bodies if the nature of the accident is such that particles of clothing, dirt, splinters, etc., may have been carried into the depths, and the incision partially closed by sutures so introduced as not to interfere with free drainage.

The only exception to this rule of treatment is in gunshot wounds of the lung, and rarely in shot wounds of the brain.

4. *Badly Contused Wounds.*—When large surfaces have been badly bruised without apparent destruction of the deep structures, the best management is to clean the skin with 65 per cent alcohol (the virtues of the "old-fashioned" tincture of arnica are ascribable to the effect of its alcohol) and then cover with several layers of gauze, either dry or saturated with the mixture of camphor and phenol above mentioned.

When there is much pain from the bruising, another phenol combination may be employed:

Chloral hydrate ......................1 part.
Phenol ..............................1 part.

Mix in a mortar sterilized by boiling and keep in a bottle similarly cleaned. Gauze or clean flannel may be saturated with this and applied to the sore. It is both antiseptic and anodyne.

Such a wound ought to be redressed in forty-eight hours in order to see that the deeper tissues are not sloughing. As soon as it is apparent that sloughing is sure to occur, it is best to open the wound freely, in several places if necessary, to permit early and free drainage. By this measure one may often prevent a deep slough and a long, tedious period of suppuration.

If in spite of the most careful antiseptic treatment constitutional symptoms arise, the treatment for the particular kind of sepsis must be instantly adopted, *i. e.*, staphylococcic, streptococcic, saphrophytic, etc.

